# Gallium SPECT/CT in evaluation of IgG4-related disease: A case report and literature review

**DOI:** 10.1097/MD.0000000000004865

**Published:** 2016-09-16

**Authors:** Tzyy-Ling Chuang, Bao-Bao Hsu, Chen-Lin Chi, Yuh-Feng Wang

**Affiliations:** a Department of Nuclear Medicine; b Department of Allergy, Immunology and Rheumatology; c Department of Pathology, Dalin Tzu Chi Hospital, Buddhist Tzu Chi Medical Foundation, Chiayi; d School of Medicine, Tzu Chi University, Hualien, Taiwan, R.O.C.

**Keywords:** case report, gallium SPECT/CT, IgG4-related dacryoadenitis and sialoadenitis, IgG4-related disease, IgG4-related sclerosing disease, IgG4-related systemic disease, Mikulicz disease

## Abstract

**Background:**

The clinical picture of IgG4-related sclerosing disease (IgG4-RSD) may mimic lymphoma, and should be in the differential diagnosis of patients with this clinical picture.

**Case summary:**

A 32-year-old female had recurrent swelling of both eyelids for more than 15 years. Examination revealed elastic, firm, swollen lacrimal glands about 2–3 cm in diameter that was not painful. Head and orbits magnetic resonance imaging (MRI) showed mass lesions over the bilateral lacrimal glands, submandibular glands, and left foramen of ovale. The differential diagnosis included lymphoid tissue, inflammatory masses, and lymphoma. Gallium single-photon emission computed tomography/computed tomography (SPECT/CT) showed uptake in the bilateral lacrimal glands, right parotid and bilateral submandibular glands, bilateral perirenal region, mediastinal, prevertebral, paraaortic, lumbar, bilateral pelvic (including internal iliac chain) lymph nodes, anterior aspect of right 3rd rib, and lateral aspect of left 6th rib. CT showed multiple enlarged lymph nodes in the mediastinum, right pulmonary hilum, prevertebral space of the thoracolumbar spine, retroperitoneal paraaortic area, bilateral parailiac areas, and bilateral perirenal spaces. Antinuclear and anti-SSA/SSB antibodies were negative, and the serum IgG4 level was 740 mg/dL (normal, 8–140 mg/dL). Right parotid gland biopsy showed abundant IgG4-positive plasma cells. Mikulicz disease (IgG4-related sclerosing disease) was diagnosed and she received glucocorticoid treatment. Follow-up CT and MRI showed with resolved eyelid swelling and perirenal mass lesions. Follow-up gallium scan was normal.

**Conclusion:**

Gallium SPECT/CT can be a useful tool for initial and follow-up evaluation of IgG4-RSD.

## 1 Introduction

IgG4-related dacryoadenitis and sialoadenitis (IgG4-DS), so-called Mikulicz disease (MD), is characterized by elevated serum immunoglobulin G4 (IgG4) and bilateral enlargement of the lacrimal and salivary glands with infiltration of IgG4-positive plasma cells, and lack of systemic inflammation.^[1,2]^ MD can present as a singular systemic IgG4-related plasmacytic disease, mean that “IgG4-related sclerosing disease or IgG4-related systemic disease (IgG4-RSD).”^[2,3]^ The disease is differentiated from Sjögren syndrome (SS) by good responsiveness to glucocorticoids, leading to recovery of gland function.^[2]^ Recent studies have indicated the importance of differentiating between IgG4-DS and malignant lymphoma.^[1]^ Characteristic patterns of gallium uptake and on positron emission tomography with [^18^F]fluorodeoxyglucose positron emission tomography/computed tomography (FDG PET/CT) scanning are helpful for diagnosis, detection of involved lesions, and differential diagnosis in patients with IgG4-related disease to avoid unnecessary surgery or incorrect treatment (such as chemotherapy).^[4,5]^

## 2 Case report

A 32-year-old female with childhood asthma has intermittent painless tense bulging of the bilateral upper eye lids for more than 15 years (since 1999). She had no diplopia, orbital pain, blurred vision, dry eyes, or dry mouth. She was informed of benign eyelid lesions in 2000 and 2002 by ophthalmologists. No biopsies were performed, and the lesions resolved with intravenous corticosteroids. However, eye lid swelling recurred after she was tapered off oral steroids. In 2003, MRI was performed, and she was diagnosed with SS. She was treated with methylprednisolone pulse therapy (MTP) for 3 days, after the eyelid swelling completely subsided. However, swelling of the upper eyelids recurred when she was tapered off oral steroids. In 2004, she had to quit her job because of recurrent eyelid swelling. In 2006, she began using Chinese herbal medicines which she stated reduced the eyelid swelling by about 50%. However, in the 3 months before being seen at our clinic, the upper eyelid swelling increased and was severe enough to produce tense bulging.

Her mother stated that she has had a cough and wheezing since her infancy, with the need for intermittent bronchodilator and intravenous corticosteroid therapy. She has no known allergies to foods or medications. Both her mother and sister have allergic rhinitis. Bilateral lacrimal gland swelling related to sicca syndrome was suspected.

**Figure 1:**
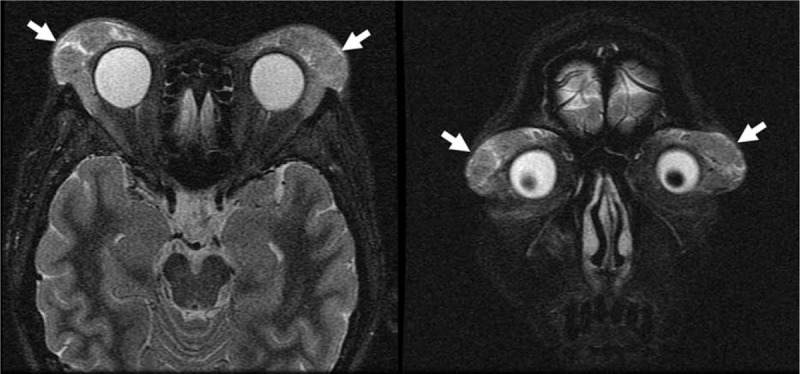
Cranial magnetic resonance imaging (MRI) revealed bilateral lacrimal gland enlargement (arrows).

**Figure 2:**
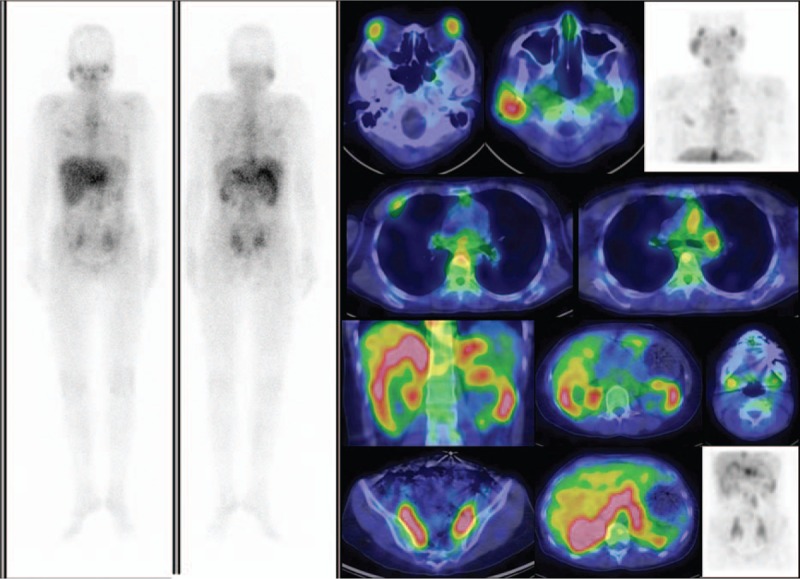
Pre-therapy gallium scan with SPECT/CT showed increased uptake in lacrimal, right parotid, and bilateral submandibular glands, and mediastinal, prevertebral, paraaortic and bilateral pelvic (including internal iliac chain) lymph nodes. There was also uptake in the 3rd and left 6th ribs, and renal and perirenal areas.

**Figure 3:**
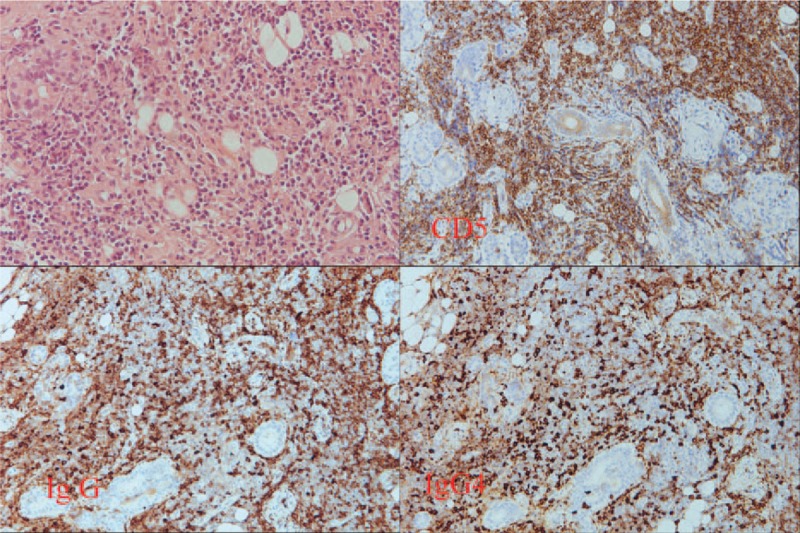
Microscopically, the section showed parotid tissue with diffuse, heavy infiltration of lymphoid and plasma cells in the stroma. Some lymphocyte infiltration into the glands and ducts was also noted. Diffuse atrophy of the parotid tissue is also found. The findings were consistent with Mikulicz disease. Immunohistochemical staining for CD5, IgG, IgG4, and kappa and lambda light chains revealed numerous IgG4-positive cells found in each high-powered field. Immunofluorescence staining for IgA, IgG, IgM, and C3 revealed IgG deposition, suggesting the possibility of IgG4-related sclerosing disease.

**Figure 4:**
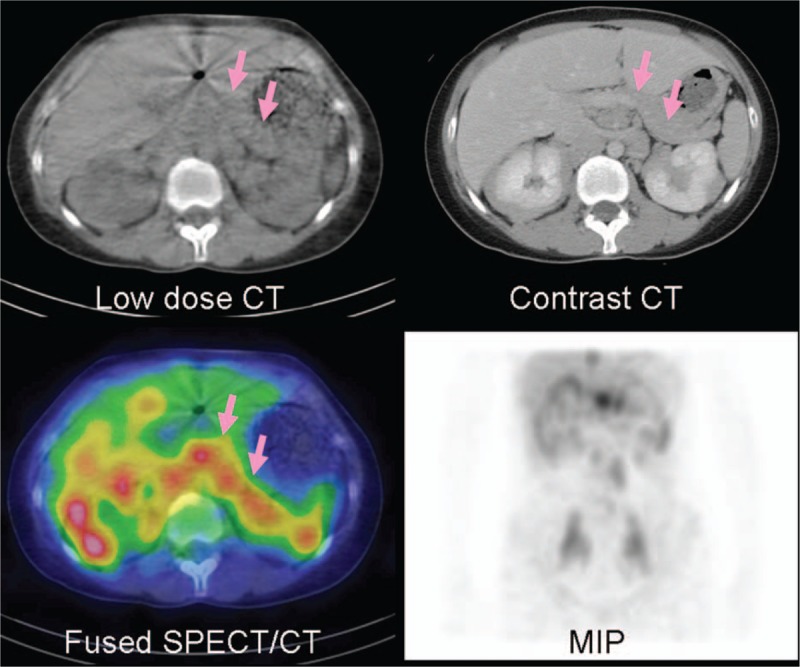
Gallium SPECT/CT revealed pancreatic involvement (arrows) of IgG4-related disease, the localization and registration were more confident as correlation with contrast CT.

A Schirmer test showed od 2 mm, os 1 mm, but the patient had no complaints of dry eyes or dry mouth. Cranial MRI revealed bilateral lacrimal gland and submandibular gland enlargement with mass infiltration into the bilateral maxillary sinuses and left foramen of ovale (Fig. 1). The differential diagnosis was lymphoid tissue, inflammatory masses, and lymphoma. Sialoscintigraphy showed a high likelihood of sicca syndrome. Immunology studies were negative for SSA/SSB, anti-nuclear antibody (ANA), anti-neutrophil cytoplasmic antibody (ANCA). In addition, elevated IgG (3790 mg/dL) and serum IgG4 (740 mg/dL), low IgM (39 mg/dL), normal IgA (163 mg/dL), and low C3/C4 (62/7 mg/dL) levels were found. A pulmonologist was consulted for suspected IgG4-related plasmacytic syndrome with lung involvement. High-resolution CT (HRCT), diffusing capacity of the lungs for carbon monoxide (DLCO), and bronchial provocation testing were performed. The DLCO and bronchial provocation tests were not consistent with bronchial asthma. HRCT showed multiple enlarged lymph nodes over the mediastinum, right pulmonary hilum, prevertebral space of the thoracolumbar spine, retroperitoneal paraaortic area, bilateral parailiac areas, and bilateral perirenal spaces (R/O lymphoma), and increased interstitial changes over the anterior right upper lobe (RUL) of the lung. Gallium scan demonstrated increased uptake in lacrimal, right parotid, and bilateral submandibular glands, and mediastinal, prevertebral, paraaortic, and bilateral pelvic (including internal iliac chain) lymph nodes. There was also uptake in the right 3rd and left 6th ribs, renal, and perirenal areas (Fig. 2). Biopsy of the right parotid gland was consistent with Mikulicz disease. Microscopically, the section showed parotid tissue with diffuse, heavy infiltration of lymphoid and plasma cells in the stroma, with some lymphocyte infiltration into the glands and ducts. Diffuse atrophy of the parotid tissue was also found. The findings were consistent with Mikulicz disease. Immunohistochemical (IHC) staining for CD5, IgG, IgG4, and kappa and lambda light chains was performed. Numerous IgG4-positive cells were noted in each high-powered field. Immunofluorescence staining for IgA, IgG, IgM, and C3 revealed IgG deposition (Fig. 3). The possibility of IgG4-related sclerosing disease was considered. Single-photon emission computed tomography/computed tomography (SPECT/CT) showed uptake to pancreatic region, indicating an additional diagnosis of autoimmune pancreatitis (Fig. 4).

**Figure 5:**
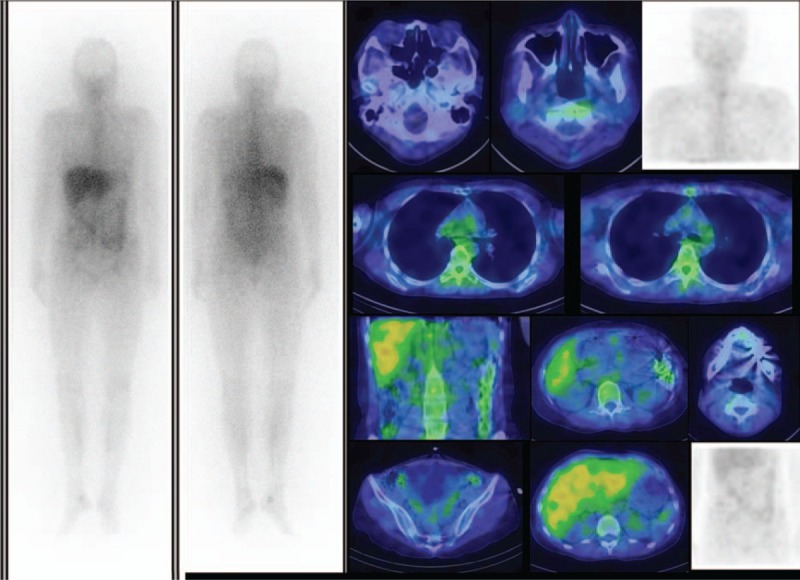
Three months after treatment, gallium scan with SPECT/CT only showed physiological uptake; the abnormal radioactive foci identified on the prior scan were no longer present.

The patient was treated with low-dose corticosteroids (methylprednisolone 40 mg intravenous) for 2 days. There was no clinical response, so MTP pulse therapy 500 mg daily for 3 days was administered. The eyelid swelling rapidly decreased, and she was able to open her eyes. Three months after treatment, gallium SPECT only showed physiological uptake; no regions of abnormal uptake were noted (Fig. 5).

## 3 Discussion

IgG4-DS has been considered to be a subtype of SS because of certain histopathological similarities, particularly lymphocytic infiltration.^[1]^ However, IgG4-DS patients show elevated serum levels of IgG4 and infiltrating IgG4-positive plasma cells in glandular tissues.^[6]^ Numerous tissues can be affected, and the pancreas, salivary or lacrimal glands, lymph nodes, kidneys, and retroperitoneum are the most frequently affected.^[7,8]^ It is considerable to distinguish IgG4-RSD from lymphoma and other similar diseases such as Castleman disease, Churg–Strauss syndrome, primary sclerosing cholangitis, secondary retroperitoneal fibrosis Wegener granulomatosis, sarcoidosis, and SS by histopathological investigation of diseased lesions.^[1]^ Japanese Society published the Diagnostic Criteria for IgG4-related MD in 2008, the criterions includes (a) persistent symmetrical swelling of lacrimal glands and major salivary glands more than 2 glands and more than 3 months; (b) high serum IgG4 levels more than 135 mg/dL; (c) IgG4-positive plasma cells/IgG-positive plasma cells more than 0.4 in the tissue by immunostaining. If any 2 of these 3 criteria fulfilled including item (a), diagnosis of IgG4-DS can be established.^[1]^ Our case was suspected for IgG4-DS because of the item (a) and (b) of criteria. For differentiating between IgG4-DS and other possible etiologies of diseases, gland biopsy was performed.

Complications of MD and various diseases encompassed by IgG4-RSD include autoimmune pancreatitis, retroperitoneal fibrosis, tubulointerstitial nephritis, autoimmune hypophysitis, Riedel thyroiditis, interstitial pneumonitis, prostatitis, lymphadenopathy, inflammatory aortic aneurysm, and inflammatory pseudotumor, all of which exhibit IgG4 involvement in their pathogenesis.^[2,9]^ Another similar entity, Küttner (KT), a chronic sclerosing sialadenitis that presents with asymmetrical firm swelling of the submandibular glands, is also associated with prominent infiltration of IgG4-positive plasmacytes.^[2]^ In MD, the benign enlargement of lacrimal and salivary glands is persistent, and secretory dysfunction is either minor or not detectable; it also lacks anti-SSA and anti-SSB antibodies.^[2]^ Sialography, sonography, and FDG PET/CT may provide information for differentiation between SS and MD.^[10,11]^ In our case, initially the lacrimal and salivary swelling, like in SS, occurred repeatedly and disappeared without treatment.^[2]^ Although steroids were effective, swelling of the lacrimal and salivary glands recurred and serum IgG4 levels increased when steroids were discontinued.^[2]^

Gallium scanning is a nuclear medicine imaging method that is used to evaluate active inflammatory processed. A study of gallium scanning focused on comparison between sarcoidosis and IgG4 disease showed uptake in different patients at variable sites including mediastinal, hilar, supraclavicular, inguinal, submandibular and paraaortic lymph nodes, lacrimal and parotid glands, lung, heart, spleen, kidney, and pancreas.^[12]^ IgG4-RSD showed uptake at various sites including hilar, paraaortic, and mediastinal lymph nodes, lacrimal, parotid, submandibular glands, lung, pancreas, and kidney.^[12]^ Thus, the 2 entities showed overlapping regions of gallium uptake. Though in our case pathological studies were not done on all of the gallium-positive lesions, all of the gallium-avid lesions in our case showed complete remission after steroid treatment. The incidental finding of autoimmune pancreatitis was also found to have resolved on follow-up gallium SPECT/CT.^[13]^ SPECT/CT also showed the uptake peripherally to the kidneys as perirenal pseudotumor, not only or instead of tubulointerstitial nephritis. FDG PET has also been studied to evaluate IgG4-RSD.^[4,5]^

Although the efficacy of corticosteroids for the treatment of IgG4-RD has been established, second- or third-line treatments are frequently required.^[3]^ IgG4-RSD relapses are frequent after treatment discontinuation. Relapses usually occur relatively rapidly, but can also occur after a long period of time.^[14]^ Involvement of more than 1 organ or lymph nodes may be associated with poor clinical outcomes.^[3,15]^

Gallium scanning with SPECT/CT and FDG PET/CT may be useful for whole body imaging for evaluation the disease distribution, disease status (active or not), localization, pretherapeutic staging, disease recurrence, therapeutic response, and treatment guidance of IgG4-RSD, and also for guiding tissue biopsy for diagnosis confirmation.^[3–5,16,17]^ Gallium scan can find clinically unfound lesions of IgG4-RSD, and probably can differential KT from MD if asymmetric salivary uptake.^[4]^ Although lacrimal uptake probably physiologic, SPECT/CT can offer the differentiation of pathological enlargement in the CT images. Pancreatic uptake can be easily recognized by SPECT/CT. The pattern of FDG uptake on PET/CT has been shown to support a diagnosis of IgG4-RSD.^[18]^ In addition, FDG PET/CT is more sensitive than conventional radiology for detecting early involvement of IgG4-RSD.^[5]^ As compared with FDG PET/CT, gallium SPECT/CT is much cheaper and more readily available, and offers similar diagnostic efficacy. Gallium SPECT/CT may also be superior to PET/CT because it allows differentiation of renal involvement (interstitial nephritis) and perirenal pseudotumor.^[19]^

Only a small number of IgG4-RSD patients present with typical clinical involvement, normal serum IgG4, typical pathological findings, and positive immunostaining.^[5]^ An increase in serum IgG4 also can be observed in several other conditions, and is thus not specific to the disease.^[20,21]^ There are also reported cases of mantel cell lymphoma presenting as IgG4-DS, MD complicated with diffuse large cell lymphoma, ocular adnexal lymphoma associated with IgG4+ chronic sclerosing dacryoadenitis, and cases developing non-Hodgkin lymphoma during follow-up of IgG4-RSD.^[1,22–24]^ Although chronic inflammation and lymphomas are causally related, the mechanism involved is still unclear.^[22,23]^

## 4 Conclusions

In our case, the initial impression after CT scan was suspected lymphoma. Biopsy of the lesion and serum IgG4 testing are essential for a definitive diagnosis of IgG4-DS. Gallium scan with SPECT/CT can identify an appropriate biopsy site, evaluate disease status and range of involvement, and treatment response. It is also a complement to other diagnostic and clinical examinations in terms of functional information. Informed consent was agreed.
